# Prediction of Promiscuous P-Glycoprotein Inhibition Using a Novel Machine Learning Scheme

**DOI:** 10.1371/journal.pone.0033829

**Published:** 2012-03-16

**Authors:** Max K. Leong, Hong-Bin Chen, Yu-Hsuan Shih

**Affiliations:** 1 Department of Chemistry, National Dong Hwa University, Shoufeng, Hualien, Taiwan; 2 Department of Life Science and Institute of Biotechnology, National Dong Hwa University, Shoufeng, Hualien, Taiwan; 3 Department of Medical Research and Teaching, Mennonite Christian Hospital, Hualien, Taiwan; University of Helsinki, Finland

## Abstract

**Background:**

P-glycoprotein (P-gp) is an ATP-dependent membrane transporter that plays a pivotal role in eliminating xenobiotics by active extrusion of xenobiotics from the cell. Multidrug resistance (MDR) is highly associated with the over-expression of P-gp by cells, resulting in increased efflux of chemotherapeutical agents and reduction of intracellular drug accumulation. It is of clinical importance to develop a P-gp inhibition predictive model in the process of drug discovery and development.

**Methodology/Principal Findings:**

An *in silico* model was derived to predict the inhibition of P-gp using the newly invented pharmacophore ensemble/support vector machine (PhE/SVM) scheme based on the data compiled from the literature. The predictions by the PhE/SVM model were found to be in good agreement with the observed values for those structurally diverse molecules in the training set (*n* = 31, *r* ^2^ = 0.89, *q* ^2^ = 0.86, RMSE = 0.40, *s* = 0.28), the test set (*n* = 88, *r* ^2^ = 0.87, RMSE = 0.39, *s* = 0.25) and the outlier set (*n* = 11, *r* ^2^ = 0.96, RMSE = 0.10, *s* = 0.05). The generated PhE/SVM model also showed high accuracy when subjected to those validation criteria generally adopted to gauge the predictivity of a theoretical model.

**Conclusions/Significance:**

This accurate, fast and robust PhE/SVM model that can take into account the promiscuous nature of P-gp can be applied to predict the P-gp inhibition of structurally diverse compounds that otherwise cannot be done by any other methods in a high-throughput fashion to facilitate drug discovery and development by designing drug candidates with better metabolism profile.

## Introduction

P-glycoprotein (P-gp), which belongs to the ATP-binding cassette (ABC) super family of transporters, utilizes the energy that is released during the hydrolysis of ATP to actively translocate a wide range of structurally unrelated compounds across the cell membrane [1]. P-gp, which is encoded by human *MDR1* (*ABCB1*) gene and localized to chromosome 7q21, can be found in a variety of normal human tissues, including liver, kidney, small and large intestines, pancreas, brain, ovary and testes [2]–[4]. It is believed that P-gp-mediated efflux plays an essential role in cellular protection as well as in secretion and/or disposition by extruding xenobiotics from mammalian cells [5]. For instance, it has been found that oral absorption and central nervous system entry of various drugs can be limited by the P-gp expression in gastrointestinal tract (GIT) and brain capillary endothelial cells, respectively [6]. As a result, P-gp exerts profound effects on the absorption, distribution, metabolism, excretion and toxicity (ADME/Tox) of an administrated drug [7].

In addition to expression in normal tissues, P-gp is also widely expressed in many human cancers, causing multidrug resistance (MDR), in which a given non-drug resistant cell or cell line becomes cross-resistant to other diverse drugs after being treated by a single drug. This will result in the reduction of intracellular drug accumulation by active extrusion of drugs from the cell [5]. For example, the efficacy of a variety of antitumor agents, such as doxorubicin, paclitaxel, etoposide and vincristine, is diminished once the tumor cells overexpress P-gp [8]. Furthermore, there is a healthy body of studies to support the fact that P-gp plays a critical role in drug resistance in infectious diseases [9], [10], brain diseases [11], rheumatoid arthritis [12] and cancers [13], resulting in impairing chemotherapeutic treatment. For instance, 17-allylamino-17-demethoxygeldanamycin (17-AAG) is the first-generation inhibitor of molecular chaperone heat shock protein 90 (Hsp90), which has been proposed to be a novel therapeutic target for a variety of cancers [14] because of its pivotal role in cancer progression and tumor survival [14]. Nevertheless, the efficacy of 17-AAG is limited by its sensitivity to MDR [15]. As such, novel Hsp90 inhibitors that can inhibit P-gp are under clinical development [16]–[18]. Thus, MDR can increase efflux of chemotherapeutical agents, reduce intracellular drug accumulation, and create a supreme hurdle in the effective chemotherapy of many disorders [19].

Inhibition of P-gp have broad and profound drug metabolism and pharmacokinetics (DM/PK) implications [20] since it can either reduce the hepatic and renal clearance or increase the bioavailability, resulting in adverse drug–drug interactions [21]. Thus, some MDR modulators may alter not only the concentration of chemotherapeutic agents in cells, but also their plasma concentrations. For example, a clinical study unequivocally demonstrated that the plasma concentration of orally administered digoxin was dramatically reduced in combination with co-administrated rifampin due to the P-gp mediated drug–drug interactions [22]. Therefore, the inhibition of P-pg plays a clinically important role in modern chemotherapy since it is hoped to find specific P-gp modulators that can efficaciously reverse MDR in resistant cell lines, restore sensitivity to chemotherapy, and thus improve treatment results [23].

**Figure 1:**
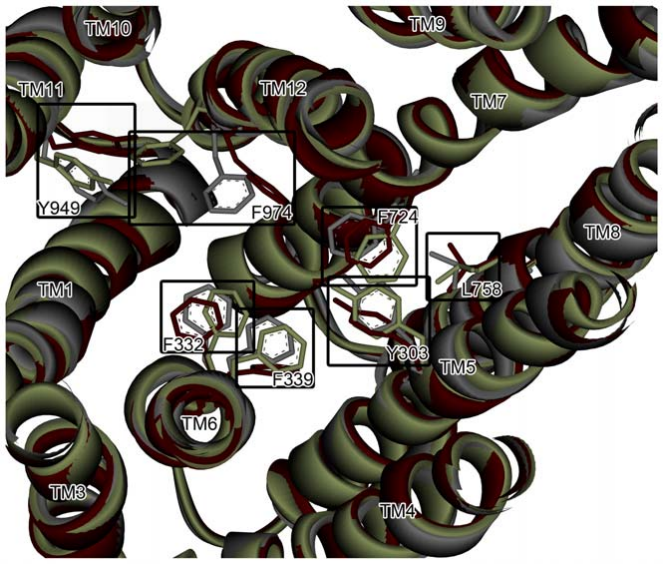
Superposed murine P-gp proteins. The superposition of three murine P-gp proteins, whose PDB codes are 3G5U, 3G60 and 3G61 and color-coded by gray, green and maroon, respectively.

*In silico* approach has been proven to be a feasible and efficient way to drug ADME/Tox assessments [24]. Of various modeling techniques, pharmacophore modeling, which develops a predictive model based on the combination of chemical features to mimic the interactions between ligands and the target protein, is often adopted [25]. In fact, numerous pharmacophore hypotheses have been proposed to predict the P-gp inhibition [26]–[33]. Nevertheless, it is believed that P-gp is a highly flexible protein [34] as manifested by the fact that it can interact with a broad range of structurally and functionally diverse compounds [35], [36]. The highly promiscuous nature of P-gp that is a common characteristic of membrane proteins [37] can be further illustrated by the published crystal structures of the bacterial lipid transporter MsbA [38] and homology models [39], [40]. Furthermore, the mouse P-pg, whose sequence shares 87% identity with human P-gp, is also highly flexible as demonstrated by Figure 1, in which the crystal structures [41], unbounded (PDB code: 3G5U) as well as co-complexed with QZ59-RRR (PDB code: 3G60) and QZ59-SSS (PDB code: 3G61), are superimposed. These proteins exhibit significant structural discrepancies, especially the amino acid residues Tyr^303^, Phe^332^, Phe^339^, Phe^724^, Leu^758^, Phe^974^ and Tyr^949^. In addition, promiscuity is not only the hallmark of P-gp conformation but also its inhibitors since it has been observed that P-gp can have multiple binding sites, *viz*. polyspecificity [42], [43], suggesting that inhibitors can interact with P-pg using different chemical features.

Accordingly, no single predictive model will suffice to accurately describe the interactions between this promiscuous protein and those highly diverse inhibitors [27], otherwise the derived predictive models can only be applied to some specific chemotypes, which, in turn, will produce substantial prediction errors once the test molecules are located outside the domain of those chemotypes. This perplexing situation can be further illustrated by the P-gp substrates, whose binding sites are blocked by most of inhibitors [44] despite of the fact that substrates and inhibitors can have different binding regions [45]. There is a growing consensus in favor of using pharmacophore ensemble to model the interactions between P-gp and substrates in order to take into consideration its promiscuous nature [46], [47], suggesting that it is plausible to accurately model the interactions between P-gp and inhibitors using pharmacophore ensemble.

Nevertheless, the promiscuous nature of P-gp and its inhibitors can be resolved using a novel scheme recently derived by Leong [48], in which a panel of plausible pharmacophore hypothesis candidates were adopted to construct a pharmacophore ensemble (PhE), which, in turn, was treated as input for regression analysis via support vector machines (SVM) and the PhE/SVM scheme can be illustrated by Figure 3 of Chen *et al* [49]. Unlike any other analog-base modeling scheme, each pharmacophore member in the PhE symbolizes a single protein conformation or a group of spatially similar protein conformations. As such, the promiscuous nature of target protein can be taken into consideration and, practically importantly, it has been shown that the PhE/SVM model executed better than the consensus prediction of multiple pharmacophore models [48] Consequently, a number of systems, whose target proteins are highly promiscuous, were also accurately modeled, including the case studies of the liability of human *ether-á-go-go* related gene (hERG) [48] as well as CYP2A6– [50] and CYP2B6–substrate interactions [51]. Additionally, the developed PhE/SVM model revealed a possible new protein conformation that was never reported before in the investigation of CYP2A6–substrate interactions [50], and it performed better than the pharmacophore ensemble [48]. The aim of this investigation was to develop an accurate, fast and robust *in silico* model based on the PhE/SVM scheme to predict the binding affinity of P-gp inhibitors. This shall facilitate drug discovery and development by designing drug candidates with better metabolism profile.

## Materials and Methods

### Data compilation

To construct quality data for this investigation, comprehensive literature search was carried out to retrieve EC_50_ values of 130 compounds, which were compiled from different source [28], [52]–[54], to maximize the structural diversity. In order to warrant a better consistency, the average values were taken in case there were two or more EC_50_ values in very close range for a given inhibitor. Furthermore, all chemical structures were examined and only those with definite stereochemistry were enrolled. All molecules assembled in this investigation and references to the literature are listed in Table S1 (Supporting Information).

### Conformation search

The conformational flexibility of studied molecules was taken into account by creating multiple conformers since three-dimensional conformations of ligands are of critical importance in developing pharmacophore models [55]. As such, all selected molecules were subjected to conformation search to generate the low-lying conformations, which were carried out using the mixed Monte Carlo multiple minimum (MCMM) [56]/low mode [57] by *MacroModel* (Schrödinger, Portland, OR). MMFFs [58] was chosen as force field and the truncated-Newton conjugated gradient method (TNCG) was set as the energy minimization method. Furthermore, the hydration effect and the solvation effect were taken into consideration by using the GB/SA algorithm [59] and water as solvent with a constant dielectric constant, respectively. The number of selected unique structures was up to 255 with an energy cutoff of 20 Kcal/mol (or 83.7 KJ/mol).

### Sample partition

The chemical and biological characteristics of selected samples in the training set play a pivotal role in determining the predictivity of a generated pharmacophore hypothesis, which can be manifested by the fact that different compound selections can produce different pharmacophore models [60]. The critical factor to constructing a perfect training set is to let *HypoGen*, which was the program employed for automatic pharmacophore generation (*vide infra*), “learn” new knowledge from the input. For examples, structurally similar compounds with significantly different biological activities or structurally distinct compounds with similar biological activities are expected to serve as perfect entries. Conversely, any redundancy in the predictive models, *viz*. overfitting or overtraining, can be yielded when structurally similar compounds with similar biological activities are selected as the training set.

Ideally, an ideal training set should consist of at least 16 molecules to warrant its statistical significance, at least 4 orders of magnitude in biological activity, approximately equal compounds in each order of magnitude and novel information concerning structure-activity relationship. More detailed selection criteria have already been discussed elsewhere [61], [62].

Thirty-one molecules, which totally consisted of 7142 conformations, were deliberately selected from all collected molecules by visually scrutinizing their chemical structures and activities to constitute the training set for automatic pharmacophore generation and regression and their associated biological activities spanned 7 orders of magnitude. The generated hypotheses were, in turn, validated by those remaining eighty-eight molecules, whose biological activities varied over 5 log units. In addition, those molecules assayed by Labrie *et al.* [63] were deliberately designated as the outlier set to assess the extrapolation capacity of the developed model, *viz*. the level of robustness, since those samples can mimic the real challenges to a predictive model in real situation. Table S1 lists molecules selected for the training set, test set and outlier set and their corresponding pEC_50_, respectively.

### Pharmacophore generation

The *HypoGen* module in *Discovery Studio* (Accelrys, San Diego, CA) was employed for automatic pharmacophore generation. It produces and ranks the pharmacophore hypotheses, which quantitatively correlate the three-dimensional arrangement of selected chemical features mapped onto those molecules in the training set with the corresponding activities through three phases, namely construction, subtraction and optimization as compared with any other QSAR techniques [64], [65], which normally rely on regression to generate predictive models. During the construction phase, *HypoGen* generates common conformational alignment among those most active molecules in the training set. The less useful pharmacophore hypotheses such as common to most inactive molecules are eliminated from the collection in the subtractive phase. The survived pharmacophore hypotheses are further improved using the stimulated annealing scheme in the optimization phase. The theory and principle of *HypoGen* have been describe in detail elsewhere [62].

Hydrogen bond donor (HBD), hydrogen bond acceptor (HBA) and hydrophobic (HP) chemical features, which depict the intermolecular interactions between an H atom on the ligand and a highly electronegative atom such as an O, N or F atom on the protein, between a highly electronegative atom on the ligand and an H atom on the protein and between nonpolar moieties on both ligand and protein, respectively, were chosen for pharmacophore hypothesis development using different feature combination and minimum, maximum and total numbers for each selected chemical feature as well as total features. In addition, the chemical feature weights and tolerances were varied in order to maximize the hypothesis diversity.

### SVM calculations

Each single predicted pEC_50_ value by those pharmacophore hypotheses in the PhE was fed as the input of SVM for further regression. In other words, those predicted pEC_50_ values were treated as descriptors for QSAR model development. As such, the dimensionality of the SVM input space corresponds to the number of pharmacophore models in the ensemble. Furthermore, the regression calculations were carried out by the SVM package *LIBSVM* (software available at http://www.csie.ntu.edu.tw/~cjlin/libsvm) using the *svm-train* module and the developed SVM models, in turn, were validated by those compounds in the test set using the *svm-predict* module. The runtime parameters, namely cost *C*, the width of the kernel function *γ* and *ε* and *ν* in case of *ε*-SVR and *ν*-SVR regression modes, respectively, were automatically scanned using an in-house perl script by the systemic grid search algorithm [66].

### Model validation

A number of statistical parameters, namely the correlation coefficient (*r* ^2^) between the predicted and observed values, standard deviation (*s*), root-mean-square error (RMSE), maximum residual (Δ_Max_) and mean absolute error (MAE) were used to evaluate the predictivity of a built model. A 10-fold cross-validation scheme, yielding the cross-validation coefficient (*q* ^2^), was also employed for internal validation.

All generated models were subjected to validations by those criteria, which were initially proposed by Golbraikh *et al.* [67] and adopted by Development of Environmental Modules for Evaluation of Toxicity of pesticide Residues in Agriculture (DEMETRA) [68], shown as follows,
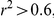


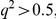


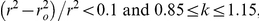


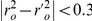
where 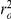 and *k* are the correlation coefficient and slope of the regression line (predicted *vs.* observed values) through the origin, respectively, and 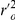 is the correlation coefficient of the regression line (observed *vs.* predicted values) through the origin.

Furthermore, the newly proposed modified version of *r* ^2^ [69], which is defined as follows,
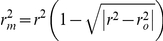
was also adopted to evaluate the quality of a predictive model, which should be large than 0.5 to be an acceptable model.

## Results

### PhE

Of all generated pharmacophore models using various selections of chemical features and runtime parameters, three hypotheses, denoted by Hypo A, Hypo B and Hypo C (listed in File S1), were assembled to construct PhE based on their prediction performances on every single molecule in the training set and test set as listed by Table S1 and their corresponding statistical evaluations as listed by Table 1. These three candidate models in the ensemble consist of a variety of combinations of chemical features, namely one HBD and four HPs in Hypo A; one HBA, one HBD and two HPs in Hypo B and one HBD and three HPs in Hypo C.

**Table 1:** Statistical evaluations, namely correlation coefficient (*r* ^2^), RMSE, maximum residual (Δ_Max_), mean absolute error (MAE), standard deviation of residual (*s*) and cross-validation coefficient (*q* ^2^) in the training set, test set and outlier set predicted by Hypo A, Hypo B, Hypo C and PhE/SVM.

	Hypo A	Hypo B	Hypo C	PhE/SVM
Training set				
*r* ^2^	0.85	0.81	0.86	0.89
RMSE	0.46	0.52	0.45	0.40
Δ_Max_	1.06	1.34	1.00	0.97
MAE	0.35	0.40	0.36	0.29
*s*	0.30	0.34	0.29	0.28
*q* ^2^	N/A†	N/A	N/A	0.86
Test set				
*r* ^2^	0.73	0.72	0.74	0.87
RMSE	0.58	0.57	0.52	0.39
Δ_Max_	1.49	1.39	1.58	1.01
MAE	0.38	0.40	0.36	0.30
*s*	0.43	0.41	0.38	0.25
Outlier set				
*r* ^2^	0.79	0.70	0.84	0.96
RMSE	0.14	0.12	0.13	0.10
Δ_Max_	0.36	0.39	0.28	0.13
MAE	0.08	0.05	0.08	0.09
*s*	0.12	0.11	0.11	0.05

† Not applicable.

**Figure 2:**
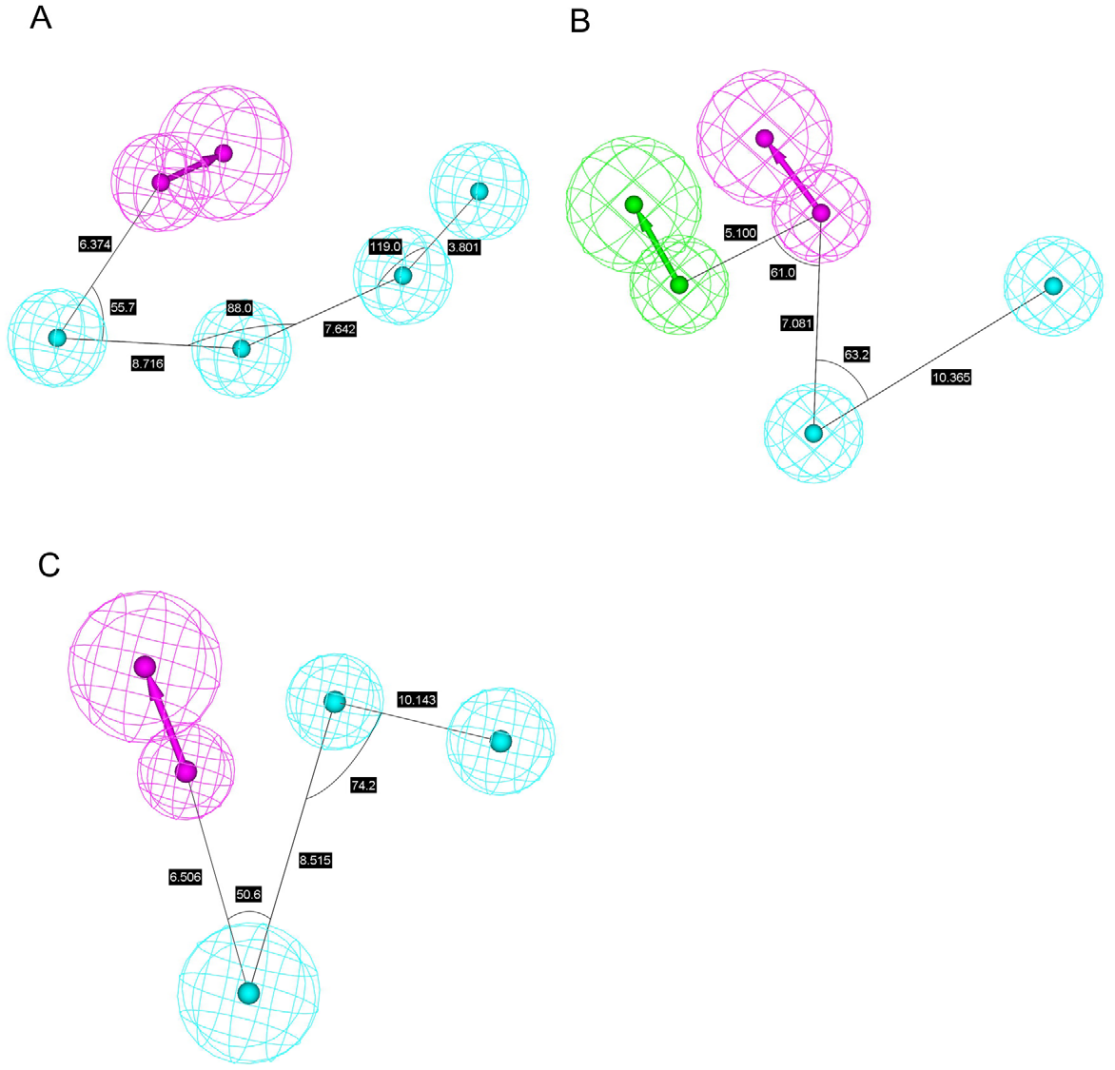
Pharmacophore models in the ensemble. Generated pharmacophore models (A) Hypo A, (B) Hypo B and (C) Hypo C, in which hydrophobic, hydrogen-bond acceptor and hydrogen-bond donor features are represented by light blue blobs, magenta blobs and arrows, and green blobs and arrows, respectively. The interfeature distances and angles among features, depicted in white, are measured in Ångstroms and degrees, respectively.

**Figure 3:**
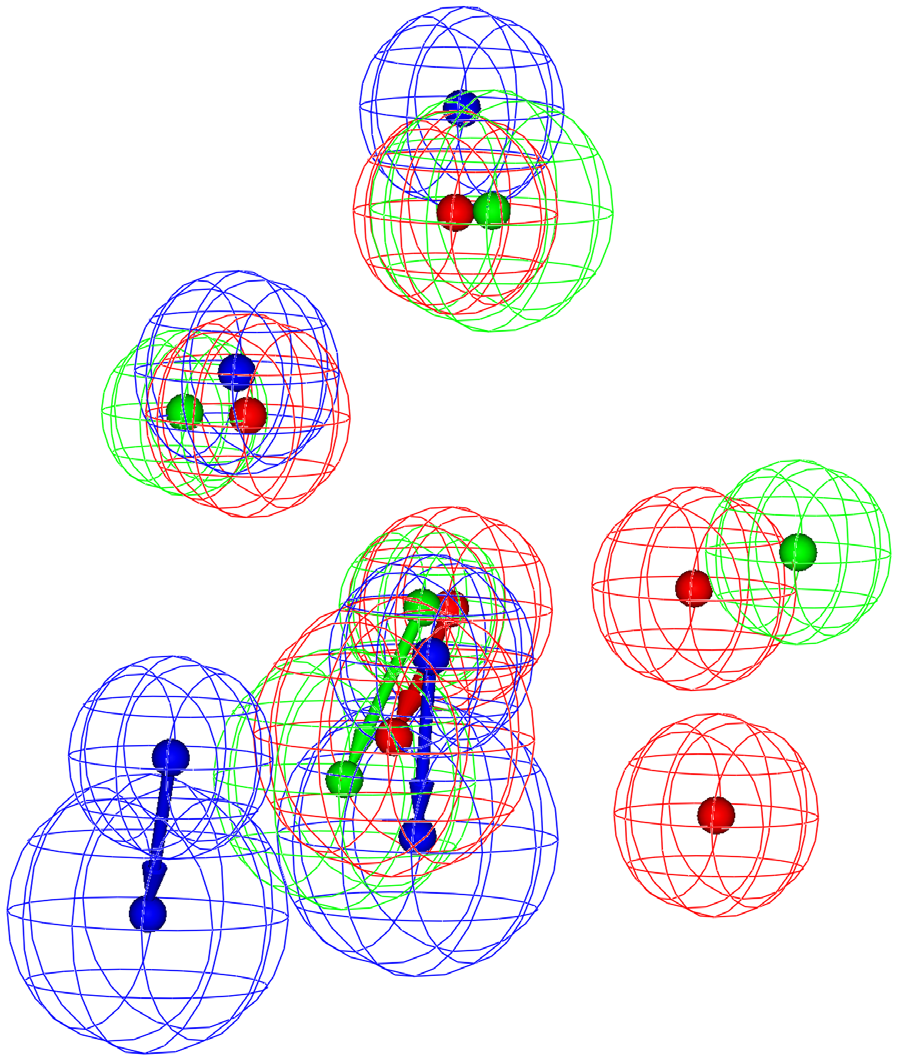
Superposed pharmacophore models. Superposition of three pharmacophore models Hypo A, Hypo B and Hypo C, denoted in red, blue and green, respectively.

In addition to various combinations of chemical features in these three pharmacophore models, their spatial arrangements are also different as exhibited by Figure 2. It can be found that one HBD and two HPs are common features among them and the closest distance between one HBD and one HP and that between two HPs are 6.374 Å and 8.716 Å in Hypo A, respectively, whereas the same measurements vary to 7.081 Å and 10.365 Å in Hypo B as well as 6.506 Å and 8.515 Å in Hypo C, respectively. The discrepancies among these three models can also be rendered by the bond angle centered at one HP and connecting to one HBD and another HP varies from 55.7° in Hypo A to 63.2° and 50.6° in Hypo B and Hypo C, respectively. Figure 3 demonstrates the superposition of these three models, and it can be observed that these three models are different not only in absolute coordinates but also in the relative relationships.

**Figure 4:**
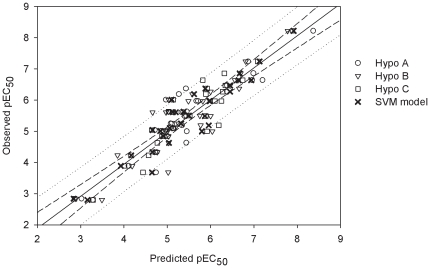
Observed *vs.* predicted pEC_50_ values in the training set. Observed pEC_50_ *vs.* the pEC_50_ predicted by Hypo A, Hypo B, Hypo C and SVM model for those molecules in the training set. The solid line, dashed lines and dotted lines correspond to the SVM regression of the data, 95% confidence interval for the SVM regression and 95% confidence interval for the prediction, respectively.

The three pharmacophore models, in general, predicted those molecules in the training set well as asserted by their less significant residuals (Table S1) and their corresponding statistical evaluations, namely parameters RMSE, MAE and *s* (Table 1). In addition, all of the correlation coefficients, *viz*. *r* ^2^ values, are larger than 0.80, suggesting their statistical significance, which can be further confirmed by inspecting the scatter plot of observed *vs.* predicted pEC_50_ values as illustrated in Figure 4.

**Figure 5:**
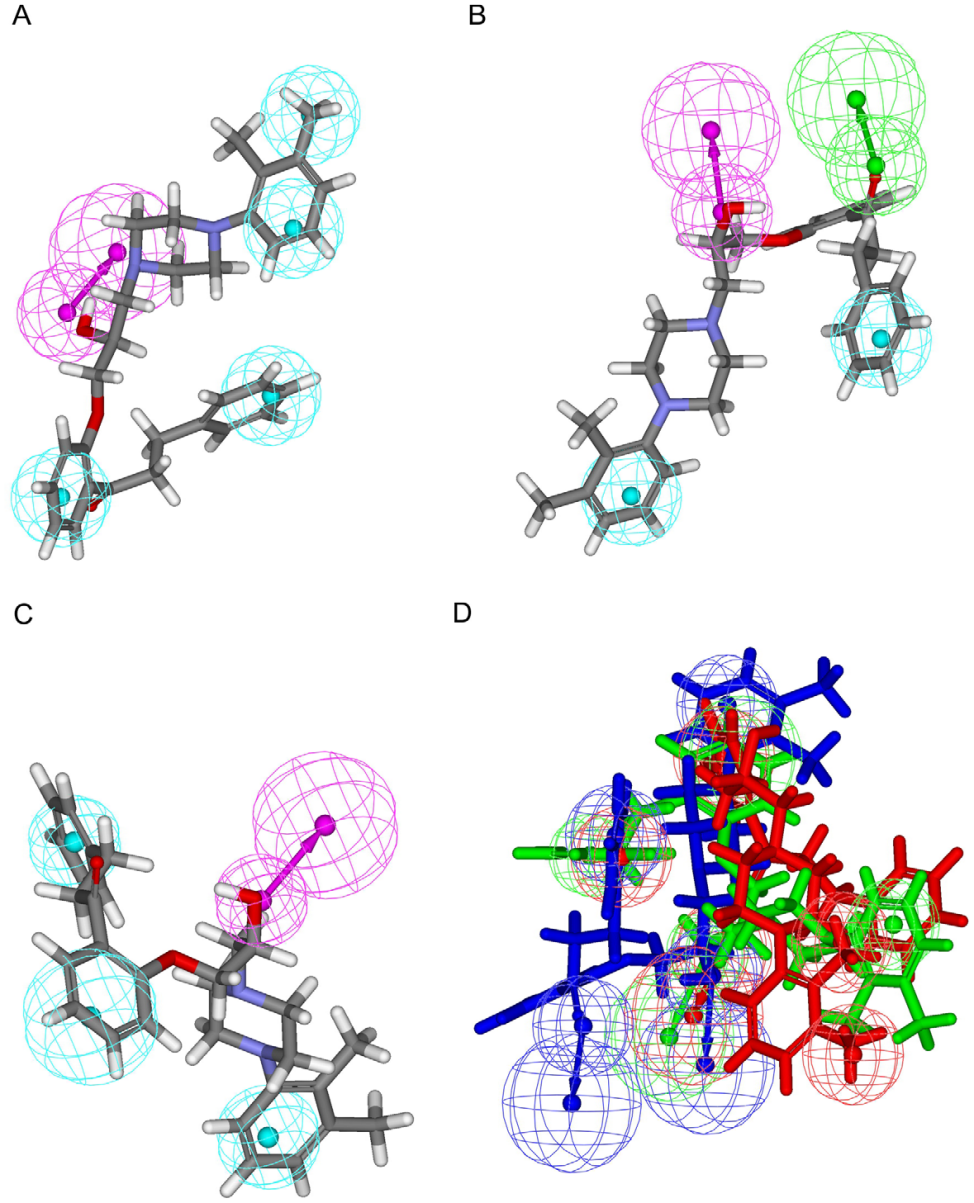
Superposition of pharmacophore models and 89. Pharmacophore models (A) Hypo A, (B) Hypo B and (C) Hypo C fitted to **89** and (E) overlay of these three models, which are color-coded by red, blue and green, respectively. The chemical features are described in Figure 2.

The maximum residuals in the training set generated by Hypo A and Hypo B were resulted from the prediction of **17** with values of −1.06 and −1.34, respectively, whose residual was only −0.76 by Hypo C. On the other hand, the prediction residuals of **50** were only −0.15 and −0.58 by Hypo A and Hypo B respectively, whereas Hypo C produced the maximum deviation of −1.00. Conversely, **84** was perfectly predicted by Hypo A, Hypo B and Hypo C with only residuals of 0.15, 0.00 and 0.13, respectively. When applied to **89**, Hypo A only yielded a residual of −0.15 and Hypo B and Hypo C showed modest errors of 0.44 and 0.33, respectively. Nevertheless, these three models adopted different conformations to bind to P-gp as illustrated in parts A–C of Figure 5, and this discrepancy becomes more pronounced by the superposition of these three conformations as depicted in part D of Figure 5, which clearly illustrates the need to construct a PhE to address the variations in protein conformation.

**Figure 6:**
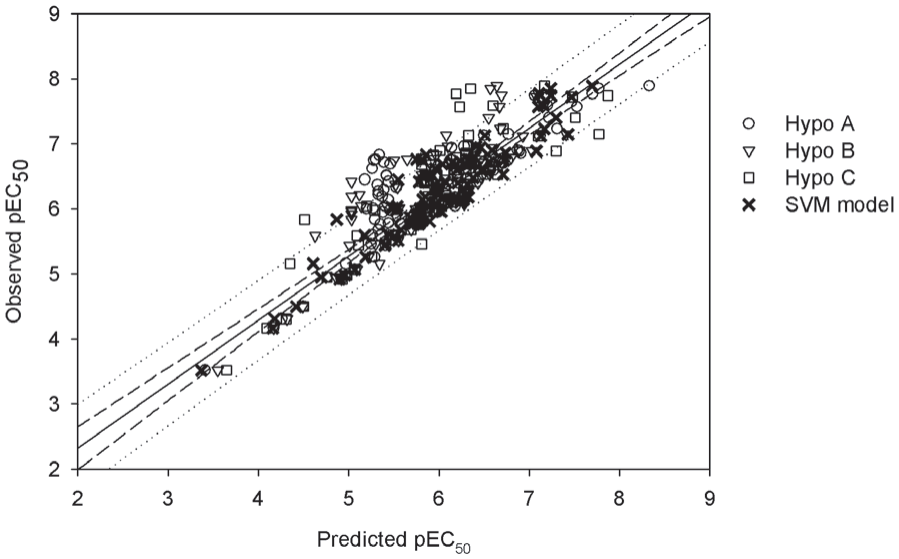
Observed *vs.* predicted pEC_50_ values in the test set. Observed pEC_50_ *vs.* the pEC_50_ predicted by Hypo A, Hypo B, Hypo C and SVM model for those molecules in the test set. The solid line, dashed lines and dotted lines correspond to the SVM regression of the data, 95% confidence interval for the SVM regression and 95% confidence interval for the prediction, respectively.

These three hypotheses in the PhE, in general, also executed well for those molecules in the test set as shown in Tables S1 and 1 and Figure 6, which displays the scatter plot of observed *vs.* predicted pEC_50_ values for those molecules in the test set. Therefore, it can be affirmed that Hypo A, Hypo B and Hypo C are qualified to constitute PhE based on the their performances in the training set and the test set as well as their statistical evaluations as mentioned above despite the fact that modest performance deteriorations from the training set to the test set can be observed as suggested by all statistical parameters. The *r* ^2^ value evaluated by Hypo A, for example, was lowered to 0.73 in the test set, *viz*. a decrease of 0.12 from the training set. Similar observations can also apply to Hypo B and Hypo C. Similar to those observations found in the training set, prediction discrepancies among these three pharmacophore models can also be found in the test set. For instance, Hypo C produced the maximum error from the prediction of **82** with an error of 1.58, whereas Hypo A and Hypo B only yielded residuals of 0.07 and 0.67, respectively. In fact, all of these three models showed various levels of overtraining, albeit marginally, as depicted by their decreases in *r* ^2^ values and other parameters from the training set to the test set.

### PhE/SVM

The final PhE/SVM model was generated by the SVM regression of those three pharmacophore hypotheses in the ensemble, yielding the number of the SVM input components (dimensionality) three. The optimal parameters for running SVM, which were selected based on the prediction results of those samples in the training set and cross-validation as listed in Table S1, are summarized in Table 2. It can be observed that the PhE/SVM model executed better than all of those individual hypotheses in the PhE for those molecules in the training set as further demonstrated by the scatter plot of observed *vs.* the predicted pEC_50_ values as shown in Figure 4, in which those points obtained from the SVM model are generally closer to the regression line than those obtained from the Hypo A, Hypo B and Hypo C. As a result, the PhE/SVM yielded the largest *r* ^2^ and the smallest RMSE, Δ_Max_, MAE and *s* among those four predictive models (Table 1). In addition, it can also be observed that the PhE/SVM model yielded residuals, which are smaller than the maximal errors produced by those hypotheses in the PhE for most of molecule in the training set and the smallest in some cases, suggesting that the PhE/SVM model is the most accurate model. The predictions of **2** by Hypo A, Hypo B, Hypo C and PhE/SVM, for example, gave rise to residuals of 0.30, 0.48, −0.28 and −0.02, respectively.

**Table 2:** Optimal runtime parameters for the SVM Model.

Parameter	Value
SVM type	*ε*-SVR
Kernel type	Radial basis function
*γ*	0.008
Cost	4
*ε*	0.001

When subjected to 10-fold cross-validation, the PhE/SVM model yielded the correlation coefficient *q* ^2^ of 0.86, which only decreased from the parameter *r* ^2^ by a value of 0.03, *viz*. a tiny difference between both correlation coefficients. Thus, it can be asserted that this PhE/SVM model exhibits highly statistical significance between the predicted values and the input data and, more importantly, it is highly possible that this SVM model is a statistically authentic model.

When applied to those molecules in the test set, PhE/SVM only shows negligible performance decreases from the training set as compared with all models in the PhE, which can be depicted by the parameters *r* ^2^, Δ_Max_ and MAE (Table 1). The MAE value, for instance, only raised from 0.29 in the training set to 0.30 in the test set despite of the fact that the sample size in the latter was ca. 2-fold more than that in the former. In fact, the parameters RMSE and *s* indicate that PhE/SVM executed better in the test set than in the training set. Thus, it can be assured that the PhE/SVM model is a better predictor than any of pharmacophore models in the ensemble for those molecules in the test set as shown by Figure 6. Most importantly, those negligible differences between both *r* ^2^ values and between *r* ^2^ and *q* ^2^ values as well as the small and consistent RMSE values in both sets manifest the fact that PhE/SVM is a well-trained predictive model since it will otherwise produce at least one substantial difference in case of overtraining.

### External validation

**Figure 7:**
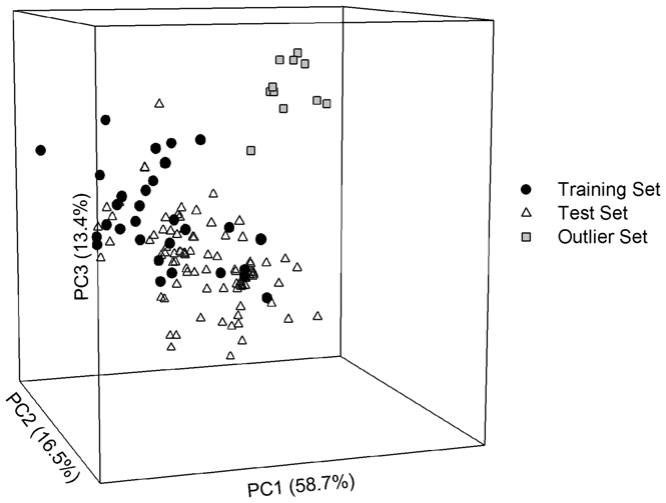
Sample distribution in the chemical space. Molecular distribution for those samples in the training set (filled circle), the test set (open triangle) and the outlier set (gray square) in the chemical space spanned by three principal components.

Eleven molecules, whose inhibition activities of P-gp were investigated by Labrie *et al.* [63], were deliberately selected as the outliers to further challenge the extrapolation power of generated models since they are completely positioned outside the perimeter of the training set in the chemical space [70], spanned by the first three principal components, which explain 88.6% of the variance in the original data, as demonstrated by Figure 7, suggesting that they serve as a good metric for the robustness evaluation of a predictive model.

The prediction results of those molecules in the outlier set are listed in Table S1 and their associated statistical evaluations are summarized in Table 1. Hypo A, Hypo B and Hypo C yielded *r* ^2^ values of 0.79, 0.70 and 0.84, respectively, in the outlier set, implying various performance decreases from the training set. Conversely, RMSE, Δ_Max_, MAE and *s* indicated that the performances of Hypo A, Hypo B and Hypo C increased from the training set to the outlier set because of lowered values of those parameters. However, this seemingly unusual characteristic for a predictive model can be realized by the fact that, of 11 molecules in the outlier set, the inhibition activities of 10 molecules are in the same log unit, *viz*. very close activities.

**Figure 8:**
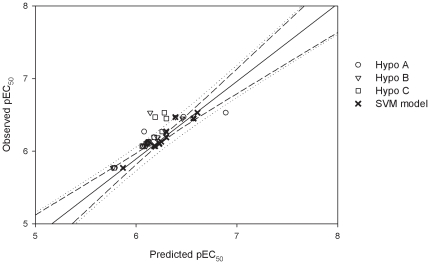
Observed *vs.* predicted pEC_50_ values in the outlier set. Observed pEC_50_ *vs.* the pEC_50_ predicted by Hypo A, Hypo B, Hypo C and SVM model for those molecules in the outlier set. The solid line, dashed lines and dotted lines correspond to the SVM regression of the data, 95% confidence interval for the SVM regression and 95% confidence interval for the prediction, respectively.

Similar to the observations found in the training set and test set, this PhE/SVM model performed better than any of pharmacophore models in the ensemble in the outlier set as indicated by those statistical parameters (Table 1) as well as the scatter plot of observed *vs.* predicted pEC_50_ values (Figure 8). Furthermore, the predictions by PhE/SVM are in extremely good agreement with observed values for all of molecules in the outlier set as manifested by the fact that the RMSE, Δ_Max_, MAE and *s* values are only 0.10, 0.13, 0.09 and 0.05, respectively, which are also smaller than their counterparts in the training set. The parameter *r* ^2^ evaluated by PhE/SVM even increased from 0.89 in the training set to 0.96 in the outlier set. These statistical evaluations assert the fact that PhE/SVM is completely insensitive to the outliers, suggesting that it is a very robust predictive model as a result, which is of pivotal importance to practical applications.

### Predictive evaluations

The predictivity of generated PhE/SVM model was further evaluated by those validation requirements proposed by Golbraikh *et al.* [67] as well as Roy and Roy [69] in the training set, test set and outlier set. The results, summarized in Table 3, indicate that PhE/SVM not only yielded high statistical values but also met all validation requirements, suggesting that this predictive model is highly accurate and predictive. Furthermore, this PhE/SVM model can maintain similar performances regardless in the training set, test set and even outlier set as depicted by the little variations among different data set. As a result, it is plausible to expect, based on the facts mentioned above, that no substantial prediction errors will be generated when applied to structurally novel compounds.

**Table 3:** Validation verification based on prediction performance of those molecules in the training set, test set and outlier set.

	Training set	Test set	Outlier set
*n*	31	88	11
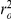	0.89	0.87	0.95
*k*	1.00	1.04	0.99
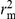	0.89	0.87	0.86
*r* ^2^>0.6	x	x	x
*q* ^2^>0.5	x	N/A†	N/A
(*r^2^*− 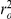 )/*r^2^*<0.1 & 0.85≤*k*≤1.15	x	x	x
| 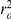 − 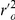 |<0.3	x	x	x
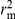 >0.5	x	x	x

† Not applicable.

## Discussion

It has been experimentally proven that P-gp has multiple binding sites [71]. As a result, Ekins *et al.* produced four pharmacophore hypotheses, which consisted of different combinations of chemical features, based on different sets of samples [30]. More importantly, the discrepancies in feature selections among these four models are consistent with the fact that Hypo A, Hypo B and Hypo C in the PhE also employed different chemical features, suggesting that different chemotypes of inhibitors can interact with P-gp using different chemical interactions, which completely agrees with the observation of Pajeva *et al.* [29], [32]. Thus, only a group of fixed chemical features, *viz*. a single pharmacophore hypothesis, cannot fully take into account the promiscuous nature of P-gp.

Furthermore, those four pharmacophore models developed by Ekins *et al.* collectively consisted of HBD, HBA, HP and ring aromatic (RA) as compared with PhE/SVM, which was collectively composed of HBD, HBA and HP, indicating that the only qualitative difference between those 4 models and PhE/SVM is the absence of RA in the latter. Statistically, the lack of RA does not deteriorate the performance of PhE/SVM as compared with those four pharmacophore models. For instance, those four predictive models generated the *r* ^2^ values of 0.77, 0.88, 0.86 and 0.76 in the training set, whereas PhE/SVM produced a value of 0.89, suggesting that the chemical feature RA is not a ncecssity to develope a predictive model. As a result, it is plausible to replace RA by HP, which can be manifested by the fact that the pharmacophore model developed by Palmeira *et al.* [33], which comprised one HBA and two RAs, predicted that the two RAs fitted onto the aromatic rings of propafenone, which, in turn, were depicted as hydrophobic by the predictive model proposed by Pajeva and Wiese [29]. In fact, none of published predictive inhibition models enrolled the chemcial feature RA except those developed by Ekins *et al.* [30], [31] and Palmeira *et al.* [33].

Furthermore, at least one HBA and one HP can always be found among all published pharmacophore hypotheses for P-gp inhibitors [26]–[29], [31], [32] except those models proposed by Ekins *et al.* [30] and Palmeira *et al.* [33]. Collectively, PhE/SVM also consisted of the chemical features HBA, HBD and HP. Nevertheless, only Hypo B adopted the chemical feature HBA among those three models in the PhE. This seemingly paradox can be understood by the fact that one of predictive models developed by Ekins *et al.* [30] did not employ the chemical feature HBA, suggesting that not all inbitors interact with P-gp using HBA. In other words, it is not necessary to always take into account HBA. As a result, it is plausible to observe that not all of hypotheses in the ensemble selected the chemical feature HBA.

**Figure 9:**
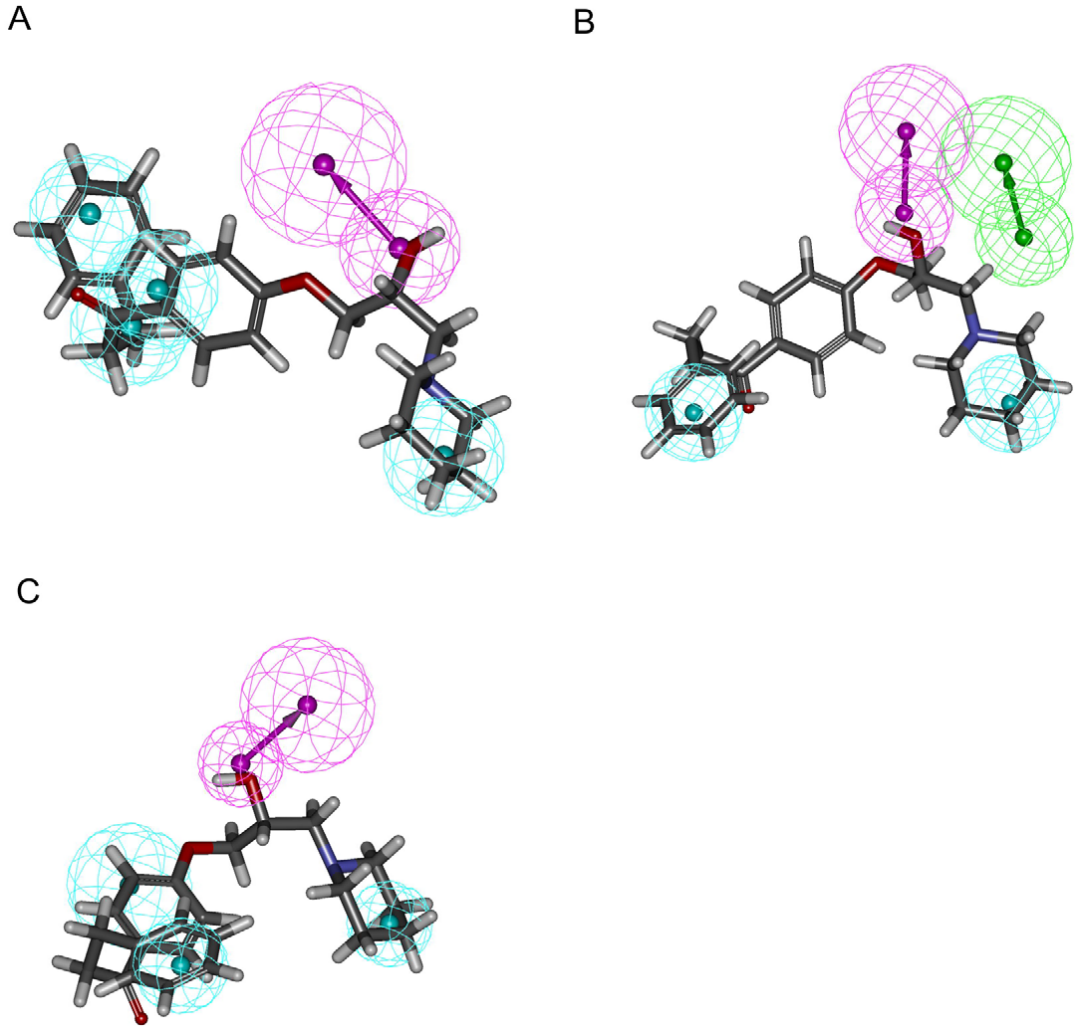
Superposition of pharmacophore models and 13. Pharmacophore models (A) Hypo A, (B) Hypo B and (C) Hypo C fitted to **13**. The chemical features are described in Figure 2.

Langer *et al.* developed a pharmacophore hypothesis, which was composed of the chemical features (aromatic) HP, HBA and positive ionizable (PI) [28]. Of 106 samples in the test set, whose experimental values were no larger than 207 µM, 34 molecules were projected as inactive since their predictive values were larger than 3,100,000 µM. These substantial discrepancies between the obseved values and predictions indicate their indiscriminations against these samples that was plausibly due to the lack of some key chemical features [28]. Conversely, the PhE/SVM model, which was collectively comprised of the chemical features HBD, HBA and HP, yielded residuals of no more than 1 log unit for those same 34 molecules, asserting that PhE/SVM is a much more accurate model and those important features were completely taken into consideration. The most pronounced discrepancy between both theoretical models are resulted from the prediction of **24**, which yielded residuals of 4.63 and 0.52 by the model derived by Langer *et al.* and PhE/SVM, respectively. Thus, it is presumable to attribute the qualitative differences between both theoretical models to the fact that Langer *et al.* enlisted the chemical feature PI without taking into account HBD, whereas PhE/SVM chose HBD over PI, suggesting that HBD plays a key role in inhibitor–P-gp interaction. The importance of HBD can be further manifested by **13**, for example, whose hydroxyl group can be perfectly fitted to the chemical feature HBD in Hypo A, Hypo B and Hypo C as illustrated by Figure 9.

In addition, numerous studies have demonstrated the importance of HBD in determining the interaction between inhibitor and P-gp. For instance, Ekins *et al.* [30] and Pajeva *et al.* [29], [32] recruited the chemical feature HBD to develop their pharmacophore hypotheses. Wang *et al.* [72], Zalloum and Taha [73] and Chen *et al.* [74] employed HBD related descriptor to construct their QSAR models; and even the CoMSIA model proposed by Labrie *et el.* [75] also used the field HBD. Accordingly, it is plausible to assume that the chemical feature HBD plays a critical role in determining the interaction between inhibitor and P-gp. Otherwise, any theoretical model may give rise to substantial prediction errors for some molecules.

### Conclusion

P-gp inhibition is vital for drug metabolism and pharmacokinetics profiling since it can lead to adverse drug-drug interactions or even toxicity. A predictive model can be greatly valuable to drug discovery and development. Nevertheless, any *in silico* model that fails to take into account the promiscuous nature of P-gp cannot accurately model the interactions between structurally distinct inhibitors and P-pg. In this study, a quantitative predictive model, derived from a novel scheme by assembling a panel of pharmacophore hypothesis candidates to construct pharmacophore ensemble, which takes into consideration protein plasticity, and support vector machine, which generates a regression model, was developed to predict the P-gp inhibition. This developed PhE/SVM showed excellent prediction accuracy for those structurally diverse 31 and 88 molecules in the training set and test set, respectively, with excellent predictivity and statistical significance. It also executed extremely well when applied to those molecules in the outlier set, which were structurally dissimilar to those in the training set, as compared with any other conventional pharmacophore models, which adopted fixed selections of chemical features and can be only used to model molecules of specific chemical structures, substantially limiting their applicability as a result. Furthermore, the PhE/SVM model can elucidate the discrepancies among all published pharmacophore models, suggesting its superiority over the other theoretical models. Thus, it can be asserted that this PhE/SVM model can be adopted as an accurate and reliable predictive tool, even in the high throughput fashion, to facilitate drug discovery and development by designing drug candidates with better pharmacokinetic profile in terms of better absorption, higher bioavailability and more efficacy.

## Supporting Information

Table S1 **Selected compounds for this study, their names, SMILES strings, observed pEC50 values and predicted values by Hypo A, Hypo B, Hypo C and PhE/SVM, data partitions and references.**
(XLS)

File S1 **Three pharmacophore hypotheses Hypo A, Hypo B and Hypo C.**
(RAR)
